# Problems and Barriers Related to the Use of mHealth Apps From the Perspective of Patients: Focus Group and Interview Study

**DOI:** 10.2196/49982

**Published:** 2024-04-23

**Authors:** Godwin Denk Giebel, Carina Abels, Felix Plescher, Christian Speckemeier, Nils Frederik Schrader, Kirstin Börchers, Jürgen Wasem, Silke Neusser, Nikola Blase

**Affiliations:** 1 Institute for Health Care Management and Research Universität Duisburg-Essen Essen Germany; 2 QM BÖRCHERS CONSULTING+ Herne Germany

**Keywords:** mobile health, mHealth, mHealth app, patient, problems, barriers, digital health applications, DiGA, app, barrier, mobile app, health care system, economic outcome, context, mobile phone

## Abstract

**Background:**

Since fall 2020, mobile health (mHealth) apps have become an integral part of the German health care system. The belief that mHealth apps have the potential to make the health care system more efficient, close gaps in care, and improve the economic outcomes related to health is unwavering and already partially confirmed. Nevertheless, problems and barriers in the context of mHealth apps usually remain unconsidered.

**Objective:**

The focus groups and interviews conducted in this study aim to shed light on problems and barriers in the context of mHealth apps from the perspective of patients.

**Methods:**

Guided focus groups and individual interviews were conducted with patients with a disease for which an approved mHealth app was available at the time of the interviews. Participants were recruited via self-help groups. The interviews were recorded, transcribed, and subjected to a qualitative content analysis. The content analysis was based on 10 problem categories (“validity,” “usability,” “technology,” “use and adherence,” “data privacy and security,” “patient-physician relationship,” “knowledge and skills,” “individuality,” “implementation,” and “costs”) identified in a previously conducted scoping review. Participants were asked to fill out an additional questionnaire about their sociodemographic data and about their use of technology.

**Results:**

A total of 38 patients were interviewed in 5 focus groups (3 onsite and 2 web-based) and 5 individual web-based interviews. The additional questionnaire was completed by 32 of the participants. Patients presented with a variety of different diseases, such as arthrosis, tinnitus, depression, or lung cancer. Overall, 16% (5/32) of the participants had already been prescribed an app. During the interviews, all 10 problem categories were discussed and considered important by patients. A myriad of problem manifestations could be identified for each category. This study shows that there are relevant problems and barriers in the context of mHealth apps from the perspective of patients, which warrant further attention.

**Conclusions:**

There are essentially 3 different areas of problems in the context of mHealth apps that could be addressed to improve care: quality of the respective mHealth app, its integration into health care, and the expandable digital literacy of patients.

## Introduction

### Background

Worldwide, patients can access an ever-growing number of mobile health (mHealth) apps. Six years ago, there were 325,000 mHealth apps available, which were created by 84,000 different developers [1]. Regardless of the high number of mHealth apps, there is no fundamental quality control by app stores as distribution channels. Thus, unsafe mHealth apps are a potential threat for patient health [2,3].

An indicator for safe mHealth apps is the approval as a medical device. This is achieved by the European conformity or Food and Drug Administration certification in Europe and the United States, respectively [4,5]. Although medical device certification focuses particularly on safety and medical-technical performance with respect to the intended purpose specified by the manufacturer, there are also other areas where problems can arise.

A more extensive certification program is the “Fast-Track Process for Digital Health Applications (DiGA)” established in Germany. In this process, in addition to the European conformity certification as proof of “safety” and “suitability,” other requirements, such as “data protection,” “information security,” “interoperability,” and “user-friendliness” are reviewed [6]. Despite the relatively broad testing approach in the fast-track process, it has not been conclusively clarified whether it is sufficient to face all potential problems in the context of mHealth apps. This is partly due to the novelty of the field of care.

In addition to governmental guidelines, there is also a variety of approaches in science to evaluate the quality of mHealth apps. Well-known assessment scales include the Mobile App Rating Scale [7], the User Version of the Mobile Application Rating Scale [8], Enlight [9], and the System Usability Scale [10]. On an aggregated level, a review by Nouri et al [11], which was published in 2018, analyzed such rating tools. The review identified “design,” “information/content,” “usability,” “functionality,” “ethical issues,” “security and privacy,” and “user-perceived value” as relevant quality dimensions [11]. However, similar to the presented governmental certification processes, it is also questionable whether these quality assessment procedures and identified quality dimensions in the scientific field are sufficient to address potential problems in the context of mHealth.

Knowing the existing problems and barriers related to mHealth apps and their use is a fundamental requirement to guarantee the comprehensive quality assurance of mHealth apps. However, only few studies have aimed to identify weaknesses and problems of mHealth apps [12-14]. A scoping review on this topic revealed 10 problem categories: “validity,” “usability,” “technology,” “use and adherence,” “data privacy and security,” “patient-physician relationship,” “knowledge and skills,” “individuality,” “implementation,” and “costs” [14].

However, it remains uncertain whether these categories are relevant from the perspective of users or patients. A systematic review of qualitative studies identified 25 studies focusing on barriers of and facilitators to the use of lifestyle apps. However, the authors emphasized that the included studies have considered only healthy individuals, and thus, the obtained results are not transferable to patients with diseases [15]. In another qualitative study from Australia, patients were recruited in a general practice setting and interviewed about barriers and enablers to the use of mHealth apps. Nevertheless, the authors emphasized that the sample size was skewed toward relatively healthy patients, and future studies should target patients with long-term medical conditions [16].

### Objectives

As there is insufficient evidence base regarding the problems and barriers in the context of mHealth apps, qualitative research methods could provide an explorative insight in this research field. Perspectives of both mHealth app users as well as nonusers should be taken into account. This is necessary to reveal existing problems with the use of mHealth apps as well as concerns and barriers preventing patients from using these apps. Therefore, this study surveyed both app using and nonusing patients in focus groups and individual interviews to identify patient-relevant problems and barriers related to the use of mHealth apps.

## Methods

We followed the standards of the study by O’Brien et al [17] and subsequently checked the manuscript by applying the 32-item COREQ (Consolidated Criteria for Reporting Qualitative Research) checklist [18] to ensure transparency in all aspects of our qualitative research.

### Theoretical Framework

Focus groups and interviews were conducted with patients who had a condition for which an approved digital health applications (DiGA) existed at the time of the study. Patients were either actual users of DiGA and other mHealth apps or did not use any mHealth apps. The aim of the research was to shed light on the perspective of patients on mHealth apps. Questions covered “problems and barriers,” “facilitating factors,” “reasons for attrition,” and “important properties” that mHealth apps should meet. This paper focuses especially on problems and barriers identified in the underlying conversations.

The decision for a qualitative research design was made to take an exploratory step into the still poorly researched field of problems and barriers in the context of mHealth apps. Conducting focus groups and interviews allowed the collection of individual opinions. Those can subsequently be used to develop new research hypotheses.

### Participant Selection

Patients were recruited via self-help groups and included both users and nonusers of mHealth apps. While patients with experience in mHealth app use could mainly contribute to the existing problems with use, patients without experience could mainly contribute to the barriers to use. Saturation was reached when all relevant indications for which there were approved DiGA at that time were covered.

Before conducting the interviews, participants received an informational letter about the topic “quality assurance of DiGA” and the proceeding of the interviews by email. Furthermore, patients were informed in the cover letter that the focus groups or interviews were part of a project (“QuaSiApps”) funded by the German Federal Joint Committee, which aims to develop a continuous quality assurance concept for DiGA. More detailed information was not provided beforehand.

### Ethical Considerations

Upon request and presentation of the project schedule to the Ethics Committee of the Medical Faculty of the University of Duisburg-Essen, it was confirmed that ethics approval was not required. This was because, as part of the patient survey, neither personal nor disease-related questions were to be asked. Written informed consent was obtained from all participants before the survey. The participants were informed and asked to consent to the recording of their conversations for subsequent transcription. At the beginning of the interviews, the participants were informed that they could end the conversations at any time without giving a reason and without facing disadvantages of any type.

### Setting

The settings of focus groups and interviews were heterogeneous. Conversations were either conducted web-based or in person. While the focus groups each included >3 participants, the interviews were conducted with 1 interviewee at a time. The onsite focus groups were conducted with self-help groups in their respective familiar settings. The web-based interviews were conducted via the videoconferencing platform Zoom (Zoom Video Communications, Inc). The choice for the web-based setting arose from the preference of participants.

No abnormalities were observed in the study setting that could influence patient statements. All patients were in a familiar environment during the interviews. No other people except 2 relatives of participants in 1 focus group were present besides participants and researchers. In 2 web-based focus groups, people did not know each other, which could lead to patients being more reluctant to participate.

There were no known relationships with participants. Notable characteristics of researchers, which could influence the participants, were not identified. There were no conflicts of interest, and the moderators did not take a firm stance for or against the use of mHealth apps.

### Data Collection

Each focus group or interview was conducted by 1 of 3 moderators (GDG, CA, and KB). Of the 3 moderators, 2 were female (CA and KB) and 1 was male (GDG). For quality assurance reasons, at least 2 of the moderators participated in each focus group or interview conducted. While 2 moderators hold degrees with health economics background (GDG and CA), 1 moderator originally stems from the field of medicine and holds an MD degree (KB). GDG and CA are researchers at the Institute for Healthcare Management and Research, University of Duisburg-Essen. KB is the managing director of BÖRCHERS CONSULTING+ and an honorary professor at the University of Duisburg-Essen. While CA and KB are very experienced in conducting focus groups, GDG was less experienced but received a detailed briefing.

A prior scoping review [14] on problems and barriers related to the use of mHealth apps similar to the German concept of DiGA served as a basis to develop a noninfluencing interview guideline. This provided a consistent interview framework (Multimedia Appendix 1). The guideline followed a uniform structure. Each topic started with open questions followed by some concrete questions, which were shown if the participants did not find a spontaneous answer. Personal data were not addressed in the guideline.

Focus groups or interviews were recorded either onsite with microphones or web-based via the integrated recording function in Zoom. Data collection took place in the period between the end of September 2021 and the beginning of December 2021. Subsequently, to record transcription, data analysis started in February 2022 and was completed at the end of October 2022. The conduct of the interviews worked without problems, and the guide proved to be understandable. Therefore, no adjustments were made to the method of data generation during the study.

The recordings of the interviews and focus groups were transcribed and pseudonymized by project assistants or employed students. A second person, also a project assistant or student assistant, performed quality assurance of the transcripts. Afterward, the transcripts were loaded into MAXQDA (VERBI Software GmbH) for data analysis, and the audio recordings were deleted. After data extraction, it was no longer possible to trace back individual statements to individual participants.

Qualitative data analysis was based on Mayring [19] and performed by GDG and CA. NB supervised the process and served as the decisive authority in the event of disagreements. As recommended by Mayring [19], deductive codes used in the data analysis were defined before the data analysis. Deductive codes were taken from the results of a scoping review [14]. Inductive subcodes were developed iteratively at the time of data extraction.

Data analysis was conducted in 4 steps to enhance trustworthiness. In the first step, GDG coded each transcript with deductive codes. In the second step, CA checked the coding made in each transcript, and in case of disagreement, problems were solved by discussion. In the third step, GDG developed inductive subcodes, which were discussed in group (GDG, CA, NB, and FP). Finally, subcodes were applied by GDG and checked by CA.

## Results

### Overview

In total, 38 patients and 2 relatives participated in 5 focus groups and 5 interviews between September and December 2021. A total of 7 conversations took place on the web via Zoom, and 3 conversations took place in person. A structured overview of the focus groups and interviews conducted is provided in Table 1.

**Table 1:** Overview of the focus groups and interviews conducted (N=40).

Number	Indication	Setting	Format	Patients, n	Date
1	Migraine	Focus group	Web-based	5	September 23, 2021
2	Tinnitus	Interview	Web-based	1	October 20, 2021
3	Obesity	Focus group	On site	10	October 21, 2021
4	Depression	Focus group	Web-based	3	October 26, 2021
5	Tinnitus	Interview	Web-based	1	October 27, 2021
6	Arthrosis	Focus group	On site	7	October 28, 2021
7	Multiple mental health disorders (depression, panic disorder, eating disorder, suicidal thoughts, sleep disorder, and agoraphobia)	Interview	Web-based	1	November 4, 2021
8	Lung cancer	Focus group	On site	8 (+2 relatives)	November 23, 2021
9	Depression	Interview	Web-based	1	November 25, 2021
10	Alcohol addiction and cancer	Interview	Web-based	1	December 7, 2021

Of the 40 included participants, 32 (80%) consented to give information about sociodemographical data (Table 2). Further details on the study participants can be found in Multimedia Appendix 2. The intrinsic motivation and participation of the participants were high. Nevertheless, the moderators made sure that everybody had their say and had the opportunity to express themselves.

**Table 2:** Core characteristics of the surveyed participants (N=32).

Characteristic			Participants, n (%)
**Sex**			
	Male	11 (34)	
	Female	21 (66)	
**Age (y)**			
	21-45	10 (31)	
	46-65	13 (41)	
	≥66	9 (28)	
**Device available to run apps^a^**			
	Any device	32 (100)	
	Smartphone	29 (91)	
	Computer or laptop	24 (75)	
	Tablet	16 (50)	
	Smartwatch	5 (16)	
**App already prescribed once**			
	Yes	5 (16)	
	No	19 (60)	
	N/A^b^	8 (25)	

^a^Multiple answers possible.

^b^N/A: not applicable.

As recruitment was conducted with the help of self-help groups, no binding statement can be made about how many participants declined to participate. None of the included patients dropped out of the study after recruitment.

The findings describe the perspectives of patients on problems and barriers in the context of mHealth apps. During the interviews and focus groups, all the 10 problem categories that served as deductive codes were addressed. The 10 mentioned problem categories, which served as deductive codes, include “validity,” “usability,” “technology,” “use and adherence,” “data privacy and security,” “patient-physician relationship,” “knowledge and skills,” “individuality,” “implementation,” and “costs.” The respective definitions of the categories can be found in the published scoping review [14]. The corresponding inductive subcodes are listed in Multimedia Appendix 3. Multimedia Appendix 4 includes all relevant statements of the patients systematized into the problem categories.

### Validity

Problems with “validity” were particularly mentioned in the 4 areas of “poor content and quality of information,” “lack of (added) value,” “lack of therapeutic setting,” and “patient safety.” The first problem area, “poor content and quality of information,” was observed by patients, especially in the lack of empirical evidence, inappropriate content, nonfunctioning links, and deficient exercise instructions:

[...] that’s why I’m very demanding when it comes to, for example, progressive muscle relaxation or something like that [...] and I find the [instructions] that are in the app-. That doesn’t work at all, so for me.

Patient with migraine

“Lack of validity, reliability, and accuracy of app-collected data” was identified as a second problem area. For example, in the area of obesity, patients reported the overrating of physical activity within the app. Another concern was that results could be skewed due to, for example, tattoos, heavy arm hair, or athletic activity, or false output could be generated by interconnectivity with other apps:

The problem is that another app somewhere is still feeding me data, even in the GoogleFit, so that I have twice the amount of steps every day. And that has blown things up, of course. I was well into the minus range of calories that I could take in. That’s not quite mature yet [...]

Patient with obesity

The third problem area of “validity” and the possible reason for the discontinuation of use is “lack of (added) value.” Patients reported that lack of health improvement would lead them to try something else:

Failure to improve health [...] “doesn’t help me, I have to try something else.”

Patient with tinnitus

The “lack of therapeutic setting” was identified as the fourth problem area of “validity.” It was explained that the app alone would not be sufficient, and a real person would have to pay attention to what the patient was doing:

With me, the problem is more. There has to be someone behind it to watch what I’m doing.

Patient with obesity

The last area to be addressed was “patient safety” under the problem area of “validity.” It was explicitly stated that the lack of human control and correction posed a direct safety issue to patients. Thus, in the context of guided practice, errors could creep in, or worse, in an emergency, such as the occurrence of suicidal thoughts, no appropriate response could occur:

Or at some point I had indicated that I was having suicidal thoughts. And only then did the app unlock that you can call the telephone counselling service directly from the app.

Patient with depression

The adverse effects that could occur through the app were also described as problematic for patient safety. For example, patients described a constant preoccupation with symptoms, symptom amplification, or the activation of triggers by the app as harmful. A final issue was the risk of social withdrawal:

Many people don’t want to talk about [the disease] and withdraw. And I think that if you only have this app in front of you and nothing personal at all, that’s very difficult for cancer patients. For all seriously ill people.

Patient with cancer

### Usability

The problems mentioned in the category of “usability” can basically be divided into “problems with the instructions” on the one hand and “difficulties with the usage” on the other. Thus, patients rejected a time-consuming introduction to the functions of the app and were dismissive toward independently searching through functions in the app:

I would say, from my experience, I really don’t have the nerve to read it at that moment. [...] But you could do it quite simply, like a how-to with screenshots. So rather with pictures where you have to click on them.

Patient with migraine

Further difficulties in the category of “usability” were found, particularly in the lack of an easy overview and nonintuitive app design. A deficient menu navigation was explicitly criticized:

But then there’s kind of no main menu after that. So when I finish it, I would like to have a main menu like that.

Patient with migraine

### Technology

In addition to the fundamental problem expressed that technical complexities are particularly disturbing in the case of illness, 3 areas of technological problems were identified: “problems with the software,” “problems with the hardware,” and “problems with interoperability and network connection.”

In the area of software, it was criticized that some apps were only available as web apps but not as mobile apps. Furthermore, patients mentioned the danger of viruses as well as problems, errors, and bugs in the software itself. Problems, errors, and bugs in the software could show up in app crashes and were seen because of poor programming:

And it was programmed in a very very clumsy and annoying way.

Patient with tinnitus

In addition to software issues, problems also arose concerning hardware, specifically with the devices in use and their updates. One point raised was related to an existing concern that updates for the smartphone would impair the functionality of the app.

Furthermore, besides updates, some technical features, such as digital displays, were criticized. Thus, for example, in the case of migraine, working on the screen was described to be problematic due to light sensitivity:

I think that I would then use it even easier if I could sort of just talk to the phone and not have to sit down and look at the display big time.

Patient with migraine

Further problems were observed with running and connected devices. Specifically, the limitation of using the app on only 1 device and the absence of the option to use it across multiple devices (such as smartphones or tablets) were considered problematic. On the other hand, additional devices, such as smartwatches and virtual reality glasses, were partially dismissed:

If you think about possible accessories, for example. I’m kind of getting out of the game with any more tech accessories. So, I don’t use a smartwatch, nor do I have VR goggles, and I don’t really want to.

Patient with cancer

In the area of compatibility and network connection, an example was cited in which data imported from other apps did not work properly. Furthermore, dependence on an internet connection was criticized:

And that I then-, we also had a bad Internet connection there, then I couldn’t use that at all like that in some cases.

Patient with tinnitus

### Use and Adherence

The problem area of “use and adherence” included, in particular, “problems due to the attitude of users”; “problems that occurred in the context of usage”; “inadequate, unappealing content design”; and “limited time resources of the patients.”

A fundamental problem stated by the patients was the attitude of users. They often have a lack of motivation and engagement. It was expressed that it may be unnecessary to prescribe apps to those who have a negative attitude in advance. On the one hand, lack of motivation may be fundamental, but on the other hand, it may be due to an aversion to the digital format:

Yes, maybe that you don’t have the motivation to do it alone. Many need the group to do the exercises.

Patient with osteoarthritis

The second that I don’t want to do therapy then on digital devices because as a journalist I’m on the go all day with tablet, cell phone and laptop.

Patient with tinnitus

If patients actually use the apps, problems can also arise in the (social) context of use. These include, for example, a low prioritization of use over other obligations such as work or private commitments, a predetermined daily schedule or paternalism exercised by the app, or distraction by other people:

And I can’t do that now in front of the running TV, when the family is sitting around, of course that doesn’t work. And if someone says: “I don’t have the time, because I have small children or we have a lot going on in the apartment or something-.” So you do need a bit of a place to retreat.

Patient with tinnitus

Furthermore, use can also be inhibited by the content design. This happens when the content is not very varied and boring or when it lacks human or emotional aspects:

For me, it was so that the app-, I didn’t find it very-, so it had become somehow boring for me. So I didn’t enjoy entering my things there anymore, because it was always the same. It always just recorded the app and then calculated it for me and that was it.

Patient with obesity

Another problem and possible reason for discontinuation of use across many indications was that apps were too extensive and time consuming. This problem was exacerbated by a lack of interruption or pause options, among other things:

It’s just very time-consuming, and that’s the thing that bothered me a little bit before.

Patient with migraine

### Data Privacy and Security

In the context of data privacy and security, fears about the loss of personal data was noted on the patients’ side. Besides insufficient data protection within the app, another problem was the lack of trust in running operating systems. Furthermore, patients rejected indiscriminate data provision to third parties:

So if I have the feeling that the data is not in good hands [...] I do turn my innermost thoughts inside out with these apps. Especially when it comes to mental illness.

Patient with depression

[...] if I now have to prove my sporting activities to the health insurance company, then they just get the sporting activities, but they are not supposed to know, how I felt [...]

Patient with obesity

### Patient-Physician Relationship

There were mainly 2 different types of problems regarding the physician-patient relationship. On the one hand, there were “problems with usage not accompanied by a doctor or therapist,” and on the other hand, there were “problems in the patient-physician relationship triggered by the app.”

A fundamental problem in the use of the app without medical or therapeutic accompaniment was seen in the limited scope of support that apps can offer. Therefore, care must be taken to ensure that they do not lead to the neglect of personal therapeutic contact:

And also to that, that an app has limits, I think that’s important and that should be apps... and that should also, when the doctors then eventually know what that is, also tell the patients [laughs], “Yes, that’s just an app and it has its limits.”

Patient with depression

Furthermore, patients expressed that app use can have an impact on the patient-physician relationship for various reasons. This occurs, for example, when patients need to report their app use to physicians or when health care providers are dismissive of the use, perceiving the app as competition to their abilities:

Because, of course, it must be assumed that the prescribing physician or the attending physician is also behind it. If he now inwardly rejects it, then it can of course be that the relationship with him suffers.

Patient with tinnitus

Finally, a particularly problematic situation for patients can arise when there are inconsistent opinions between the physician and the app. Patients then find themselves in the situation of having to determine which information is correct:

Do I trust my doctor more and the statement he says? Or do I also additionally trust what an app suggests I might do? That’s where I stand in between.

Patient with cancer

### Knowledge and Skills

Problems in the area of “knowledge and skills” were seen on the “patient side” but also on the “side of physicians and therapists.” On the part of the patients, a distinction was made here between actual “lack of skills, knowledge and experience” on the one hand and “perception” on the other hand.

The respondents saw potential for improvement particularly in the areas of patients’ technical skills and media competence. Although in the case of media competence, the assessment was seen as particularly problematic, in the technical area, the focus was on the lack of skills:

I can’t operate at all, I can’t operate at all. I’m not a person who can handle digital things at all.

Patient with cancer

Participants expressed that older patients do not always consider themselves as a target group. Older adults often had a preference for analog methods:

[...] I’ve already handed over the 60, we just didn’t grow up with these things in our hands. So maybe we just don’t want to be digital for a change, and the grip on paper is just more familiar.

Patient with migraine

In some cases, patients did not use apps because there was a lack of confidence in the therapeutic effect, the apps were perceived as complicated, or patients were fundamentally opposed to the technology:

I haven’t used an app until now, for the reason that I always imagined it to be very complicated, I have to read a lot, scroll.

Patient with obesity

Issues regarding “knowledge and skills” were also observed on the practitioners’ and health insurers’ side, particularly stemming from a poor level of knowledge:

I think it would make a lot of sense. So you would just have to somehow bring it to the doctors a bit, because I think most of them don’t really know how to deal with it yet.

Patient with depression

### Individuality

A further group of problems and a possible reason for discontinuing use was observed in the lack of “individuality.” In particular, “inadequate adaption to individual user abilities and needs” as well as the problem of “too generalized approaches” were addressed. It was emphasized that individual abilities are different, older users have special requirements for app use, and preferences exist for different (physical and mental) exercises:

Is so individual, of course. Everyone has their own problems of course, everyone perhaps does their own exercises.

Patient with osteoarthritis

Approaches that are too generalized were considered a further problem in this category. Depending on the stage of the disease and the individual, different configurations or designs were desirable:

Tinnitus symptoms are very individual, both in terms of the triggers and the expression. And dealing with it and then the question is always, if such an app comes as a therapy concept, how fine-tuned is that [...]”

Patient with tinnitus

### Implementation

The field of “implementation” included many different problems. The focus was particularly on the “barriers to access” and “additional burden for patients” as well as “additional burden for health care providers” and “low acceptance by health care providers.” Other points mentioned were “difficult transfer into clinical practice,” “too many options to use,” and “fear of consequences due to app usage.”

Patients encountered barriers to access, especially due to language barriers and lack of accessibility. The individual degree of illness and age should also be taken into account in the context of access. Further barriers to access were seen in complicated access to the app or complicated acquisition via the health insurance company and use limited to 1 device:

So, yes, it has to be low-threshold and if it’s not, I think it’s also difficult from a certain age then to deal with it or also from a certain degree of illness.

Patient with depression

The interviewees stated that the use of apps was associated with additional effort, both for themselves and for the physicians involved. In particular, the time required was emphasized critically:

Because that’s another new task for the doctors. First of all, they have so much to do with their patients, who are all individuals, focused on their specialty. So it becomes difficult to bring it all together afterwards. Because there won’t be just one patient who wants to use such an app, but five or even 100.

Patient with cancer

Patients stated that problems also arise when service providers only have a low level of acceptance toward the apps. This can be due to a lack of interest, competition between the app and health care providers, or a fundamental rejection. Physicians might perceive apps as interfering with their expertise, might express disapproval, or might advise patients against using apps. Divergent opinions between different physicians were mentioned as a particular problem:

Do I go to my urologist, do I go to my thoracic surgeon, do I go to my family doctor, or do I go to my dermatologist? And if one is in favor, but the others are against, what do I do?

Patient with cancer

“Difficult transfer into clinical practice” was identified as a further problem. The transfer might be impeded by a lack of presence of the apps. It was criticized that the apps are still rarely recommended by health care providers and that the social awareness is still low:

Because just no one has recommended this app yet. So I’ve really been through a bunch of psychologists and I-, several clinics [...] But the topic of the app is not in the waiting room with a flyer, nor at the doctor’s office, nor in the support group [...]

Patient with depression

The confusing app market was also described as complicated. Especially for patients who are already limited by diseases, the search and selection process can be complicated and time consuming:

I’m struggling with my illness or I’m struggling with my various illnesses, and then now there’s this additional psychological pressure: “I might have the wrong thing I’m using after all. Maybe there’s something significantly better in the meantime.” These digital health apps, they’re totally confusing to me as a user.

Patient with cancer

A final, but not specified, problem was seen in the consequences, especially for the health insurance of patients. It was questioned whether using the apps might have any consequences on their insurance coverage:

Then what does that do to my health insurance coverage afterward?

Patient with cancer

### Costs

Patients stated that costs might result in different problems. Patients, health insurers, and physicians were named as the groups of people affected by costs. Problems were observed in “loss of revenue,” “low willingness to pay,” “alternative financing methods,” and “waste of money.”

While a loss of revenue was considered problematic on the side of the service providers, a low willingness to pay was observed on the side of the patients. The lack of willingness to pay was related, on the one hand, to the apps themselves and, on the other hand, to the accessories required:

So for me, the reason was that it’s paid now and that-, so this app was super good, except for some initial difficulties, but just, I didn’t want to pay for it.

Patient with obesity

Alternative financing methods, especially through advertising, were another problem mentioned. Both advertising for products within the app and advertising about the app in other media were discussed:

If, for example, advertising were to come on all at once. If now from different drug manufacturers there is always something inserted there.

Patient with cancer

The last point mentioned in the category of costs was that under certain circumstances, mHealth apps could lead to a waste of money. Two such situations were discussed. First, a lack of control over whether the app is used was considered critical; second, payment, despite a lack of evidence, was criticized:

And it was, that was also point of attack, of those who do not think anything at all of e-health, that the scientific proof is missing and now the insurance already pays.

Patient with tinnitus

## Discussion

### Principal Findings

Our study showed that patients face a multitude of problems and barriers in the context of mHealth apps. While the problem categories were determined based on a previously conducted scoping review [14], many new expressions of problems emerged in each category. These expressions provide a deeper insight into the thinking and attitudes of patients related to mHealth apps.

There were mainly 3 different factors related to problems and barriers in the context of mHealth app use. First, the mHealth apps themselves could lead to problems. Such problems were observed, for example, in defective design, technical aspects, or low or no added value for patients.

Second, the integration into the health care system was considered partially problematic. Thus, problems were found, for instance, in remuneration and lack of time of health care providers, influences on the relation between health care providers and patients, and a lack of presence of the topic in the health care system.

Third, the users themselves as well as their attitude and knowledge were found to be a barrier for mHealth app use. Several participants expressed a lack of interest in using mHealth apps and explained that they sometimes lack knowledge and skills to use the digital technology. This fact was especially pronounced for older patients.

In our study, we included patients with different diseases (migraine, tinnitus, obesity, depression, arthrosis, cancer, and alcohol addiction) to receive broad feedback from people with diverse needs. While most of the problems were overarching and concerned the general use of mHealth apps, some problems were disease specific. The latter were mainly found in the category of individuality but also included technological problems such as light sensitivity leading to an avoidance of screen use in the case of patients with migraine or other problems such as the risk of increased social withdrawal of individuals with cancer because of app use. For other indications, there are additional disease-specific problems that could not be completely covered here.

### Implications

To guarantee a sustainable, safe, and effective use of mHealth apps, it is necessary to deal with all three areas where problems can arise or barriers exist: (1) the mHealth apps, (2) the integration into the health care system, and (3) the users.

#### mHealth Apps

Problems with the mHealth apps themselves were found in the categories of “validity,” “usability,” “technology,” “individuality,” “data privacy and security,” and “costs.” One approach to identify these is to use various quality assessment tools, which aim to guarantee a high quality of mHealth apps [11,20,21]. Quality assessment tools provide a practicable approach to distinguish high-quality mHealth apps from low-quality mHealth apps and thereby indicate if patients might have more or less problems with app use.

Furthermore, patients should be involved during the whole development process of mHealth apps. This includes (1) the need assessment, (2) the design and development, (3) the laboratory evaluation, and (4) the field evaluation. Eligible methods include qualitative data collection methods such as interviews, focus groups, observations, and think-aloud techniques as well as quantitative approaches such as self-report questionnaires [22].

An eligible way to do this is the participatory design [23]. By this, patients would have the possibility to comment on problems and barriers as soon as they become apparent. By including both experienced and nonexperienced mHealth apps users during the development process of mHealth apps, problems and barriers can be minimized for all users. A good example in which a 3-phase user-centered design approach has been successfully implemented is the study by Newton et al [24].

In addition, problems and barriers become apparent through user feedback in app stores. Therefore, user feedback should not only be included during the development but also in the evaluation and rework of mHealth apps. One innovative approach to incorporate user text review is the ACCU^3^RATE rating scale [25].

#### Integration of mHealth Apps Into the Health Care System

The results of this study show that problems and barriers are not restricted to the mHealth apps themselves. Thus, it is necessary to obtain more knowledge about the contextual problems and barriers that patients encounter when they want to or actually use mHealth apps.

The focus groups and interviews conducted indicate that, in the perception of patients, health care practitioners are one of the most important stakeholder groups facing problems with mHealth apps. Therefore, it is a fundamental requirement that they are convinced of the positive effects and low risk of mHealth apps for their patients and of the simple and sustainable integration in their daily routine.

However, this is not always the case, as shown by a study on the attitudes of physicians toward mHealth apps falling under the German concept of DiGA [26]. In principle, general practitioners, other outpatient care physicians, and psychotherapists were in favor of prescribing DiGA but faced significant barriers, such as insufficient information, insufficient reimbursement for DiGA-related medical services, lack of medical evidence, and legal and technological uncertainties. Therefore, such problems and barriers faced by health care providers should also be further investigated.

Germany was the first country in the world to incorporate certain mHealth apps (ie, DiGA) as a fixed part into the benefit package of the health care system [26]. Although the German concept is an innovative and commendable approach, it still faces several challenges. Further investigations into problems and barriers related to integrating mHealth apps could provide insights into improving regulatory systems. This would aim to make app use easier while ensuring safety and sustainability.

Another problem, depending on the respective health care system, could be the costs of using mHealth apps for patients. In the German statutory health insurance system, however, the costs are covered and thus represent only an indirect problem for users. Therefore, prices must be negotiated between health insurers and manufacturers, in particular.

#### mHealth App Users

Besides the mHealth apps themselves and their integration into health care, problems on the patients’ side were also reported. In our study as well as in the literature [14], it was found that low digital literacy of patients was seen as a problem in the context of mHealth app use.

According to the technology acceptance model by Davis et al [27], an actual system use is the result of perceived ease of use as well as perceived usefulness. Therefore, another approach to optimize the use of mHealth apps should focus on reinforcing the digital literacy of potential users, especially older patients. This involves supporting them with app use and effectively communicating the benefits of mHealth apps [28,29].

Both our study and other studies [14,16,30] found that age is a problem in the context of mHealth app use. Interestingly, however, the reason for this is predominantly seen in the lack of technological affinity and digital literacy among older people. The extent to which cognitive and psychological decline plays a role should be investigated further.

### Limitations

Given the scarce evidence regarding problems and barriers in the context of mHealth app use, qualitative research seems to be an eligible first step to gain further evidence [14]. Nevertheless, qualitative research is always accompanied by uncertainty. The statements made by participants are not necessarily representative for all patients, especially as participants were recruited from self-help groups and presumably had a high motivation to actively shape their to actively shape their experience of living with the disease. Thus, the problems and barriers identified in this study should serve as the first evidence to conduct further qualitative and quantitative studies.

Two points concerning the methods must still be made. First, we were not able to determine the number of people who refused to take part in our study. As we recruited our participants via self-help groups, we do not exactly know how each group distributed the information about our study to their respective members. Second, we did not calculate an agreement rate or other measurements regarding the coding discrepancies. In retrospect, however, there were very few disagreements and minimal need for discussion in this regard.

To make the research comprehensible and as free from arbitrariness as possible, we described the methods precisely. Therefore, we followed the standards by O’Brien et al [17] and checked the manuscript against the 32-items of the COREQ checklist [18]. Thereby, the strengths and weaknesses of our study became very transparent.

In Germany, the use of mHealth apps in the context of diseases is not yet very common [31]. This also became obvious in our study. Only 16% (5/32) of the participants in our study reported ≥1 prescription of an mHealth app. Thus, most participants could not contribute with experience in this field. Nevertheless, we included opinions, fears, and concerns of nonusers in our research. These should be taken into account when developing and integrating mHealth apps in health care.

A limitation of the article, but not of the study or the results, is that the patient statements were originally made in German and subsequently translated into English. We have taken care not to change the meaning of the statements. The original citations can be requested from the authors of the study.

### Conclusions

Problems and barriers in the context of mHealth apps should be considered to guarantee their sustainable, safe, and effective use. Such problems can be categorized into problems originating from the app, problems with the integration of the mHealth app into the health care system, and problems and barriers on the users’ side. While problems on the level of the app and the health care system should be taken into account when developing mHealth apps and corresponding assessment tools, problems on the patients’ side should be solved by increasing the digital literacy of potential users. On the basis of our findings, further research should be conducted to generate more evidence on problems and barriers as well as how to counteract them.

## Appendix

Multimedia Appendix 1 Interview guidelines.

Multimedia Appendix 2 Participant characteristics.

Multimedia Appendix 3 The coding system including problem categories and subthemes.

Multimedia Appendix 4 Systematized statements of the patients.

## Abbreviations

COREQ: Consolidated Criteria for Reporting Qualitative Research
DiGA: digital health applications
mHealth: mobile health

## Data Availability

All data generated or analyzed during this study are included in this published article and its supplementary information files. In addition to the English patient statements included in the text, the original statements made in German can be requested from the authors.
