# Supplementary material for: Problems and Barriers Related to the Use of mHealth Apps From the Perspective of Patients: Focus Group and Interview Study
Source: J Med Internet Res. 2024 Apr 23;26:e49982. doi: 10.2196/49982 (PMC11077409; doi:10.2196/49982)
Supplement: Multimedia Appendix 1 [file jmir_v26i1e49982_app1.docx]

## Appendix 1: Interview Guidelines

| Introductions and opening question | |
| --- | --- |
| **Open question** | **Concretization questions** |
| 1. "Spontaneously, what are the most important features that a health app has to fulfill, in order for you to   … use the app at all?  … use the app regularly / in the long term?  What makes a good DiGA?" | x |
| Problems with the supply of DiGA | |
| **Open question** | **Concretization questions** |
| 1. "What would / could argue against the use of DiGA from your point of view and experience?" | "Would  … data protection…  … lack of technical skills…  … confidence in the DiGA / scepticism…  … concerns of the physician regarding the responsibility for risk…  be possible reasons?" |
| Reasons for discontinuation of use | |
| **Open question** | **Concretization questions** |
| 1. "From your perspective and experience, what could lead to a discontinuation of DiGA use?" | "Would  … lack of technical skills…  … time consumption…  … lack of health improvement…  … social context…  be possible reasons?" |
| DiGA properties | |
| **Open question** | **Concretization questions** |
| 1. "What features would the DiGA need to have in order to be user-friendly from your point of view?"   "Think also of possible accessories (e.g. smartwatches, VR glasses)" | How important is it for you  … that the DiGA is easy to use?  … the layout of the DiGA?  … navigation through the DiGA?  … instructions or support for use?  … that the DiGA is available in several languages? |
| 1. "How do you envision an individualized DiGA?"   "Should it be possible for you to make user-specific adjustments in the DiGA or for the DiGA to respond individually to your input?" | X |
| 1. "How do you rate the statement: “When using a DiGA, my relationship with my treating physician suffers.”"   "In this context, would it be important for you to talk to your doctor about the use and results of the DiGA?" | X |
| End | |
| **Open question** | **Concretization questions** |
| 1. "Open points?"   "Further comments?" | X |
