# Supplementary material for: Problems and Barriers Related to the Use of mHealth Apps From the Perspective of Patients: Focus Group and Interview Study
Source: J Med Internet Res. 2024 Apr 23;26:e49982. doi: 10.2196/49982 (PMC11077409; doi:10.2196/49982)
Supplement: Multimedia Appendix 2 [file jmir_v26i1e49982_app2.docx]

## Appendix 2: Participants characteristics

| Characteristic | | Participants, N (%) |
| --- | --- | --- |
| Gender | | |
| Male | | 11 (34.4%) |
| Female | | 21 (65.6%) |
| Age, yrs | | |
| 21-25 | | 1 (3.1%) |
| 26-30 | | 4 (12.5%) |
| 31-35 | | 2 (6.3%) |
| 36-40 | | - |
| 41-45 | | 3 (9.4%) |
| 46-50 | | 1 (3.1%) |
| 51-55 | | 4 (12.5%) |
| 56-60 | | 4 (12.5%) |
| 61-65 | | 4 (12.5%) |
| 66+ | | 9 (28.1%) |
| Size of the place of residence | |  |
| X ≤ 5,000 | | 0 |
| 5,000 < X ≤ 20,000 | | 3 (9.4%) |
| 20,000 < X ≤ 100,000 | | 10 (31.2%) |
| X > 100,000 | | 18 (56.2%) |
| n.a. | | 1 (3.1%) |
| Education | |  |
| Secondary school: from year 5 to 9 | | 5 |
| Secondary school: from year 5 to 10 | | 11 |
| University-entrance diploma from year 5 to 12/13 | | 16 |
| Device available to run apps (Multiple answers possible) | | |
| Any device | | 32 (100%) |
| Smartphone | | 29 (90,6%) |
| Computer/Laptop | | 24 (75%) |
| Tablet | | 16 (50%) |
| Smartwatch | | 5 (15.6%) |
| Previous use of mHealth apps | | |
| Yes | 16 (50%) | |
| No | 15 (46.9%) | |
| n.a. | 1 (3.1%) | |
| mHealth app already prescribed once | | |
| Yes | | 5 (15.6%) |
| No | | 19 (59,4%) |
| n.a. | | 8 (25%) |
