# Supplementary material for: Problems and Barriers Related to the Use of mHealth Apps From the Perspective of Patients: Focus Group and Interview Study
Source: J Med Internet Res. 2024 Apr 23;26:e49982. doi: 10.2196/49982 (PMC11077409; doi:10.2196/49982)
Supplement: Multimedia Appendix 3 [file jmir_v26i1e49982_app3.docx]

## Appendix 3: Coding system including problem categories and sub-themes

| **No.** | **Problem category (=Deductive codes)** | **Sub-codes (=Inductive codes)** |
| --- | --- | --- |
| 1 | Validity | - Poor content and quality of information - Lack of validity, reliability and accuracy of app-collected data - Lack of (added) value - Lack of therapeutic setting - Patient safety |
| 2 | Usability | - Problems with the instructions - Difficulties with the usage |
| 3 | Technology | - Problems with the software - Problems with the hardware - Problems with interoperability and network connection - Unspecific technical problems |
| 4 | Use and adherence | - Problems due to the attitude of users - Problems that occurred in the context of usage - Inadequate, unappealing content design - Limited time resources of the patients |
| 5 | Data privacy and security | - Lack of data security and data protection |
| 6 | Patient-Physician relationship | - Problems with usage not accompanied by a doctor or therapist - Negative influences on the doctor-patient relationship - Divergent opinion between doctor and app |
| 7 | Knowledge and skills | - Problems on patient side   - Expandable skills, knowledge and experience of patients   - False perception - Expandable skills, knowledge and experience of health care providers |
| 8 | Individuality | - Inadequate adaption to individual user abilities and needs - Too generalized approach |
| 9 | Implementation | - Barriers to access - Additional burden - Low acceptance by health care providers - Difficult transfer into clinical practice - Too many options to use - Fear of consequences due to app usage |
| 10 | Costs | - Loss of revenue for health care practitioners - Low willingness to pay - Alternative financing methods - Waste of money |
