# Supplementary material for: Problems and Barriers Related to the Use of mHealth Apps From the Perspective of Patients: Focus Group and Interview Study
Source: J Med Internet Res. 2024 Apr 23;26:e49982. doi: 10.2196/49982 (PMC11077409; doi:10.2196/49982)
Supplement: Multimedia Appendix 4 [file jmir_v26i1e49982_app4.docx]

## Appendix 4: Systematized statements of the patients

| **Validity** | |
| --- | --- |
| **Poor content and quality of information** | - "[…] that's why I'm very demanding when it comes to, for example, progressive muscle relaxation or something like that […] and I find the [instructions] within the app-. That doesn't work at all, at least for me." (Migraine) - "But besides what I said, I would also find it important that the data is actually maintained. Meaning, that I don't eventually come across a link that doesn't work." (Obesity) - "That exercise programs are perhaps also adapted a bit to the season. I don't want an app telling me in summer I should go skiing for 30 minutes, please." (Obesity) - "what's the point that they were listed and then when you did a little bit of research you found out, "oh, they're in this list for 1-2 years and can be prescribed and the proof of the scientific evidence is still ongoing [...]. I found that a bit strange." (Tinnitus) |
| **Poor validity, reliability and accuracy of data collected by the app** | - "Whereas the exercise, it was very exaggerated. So that was completely utopian, if you walked a little bit, they were taking thousands of calories off there." (Obesity) - "But that maybe you don’t always perceive that to be super verified. Because in one article it said, for example, that if you have tattoos or heavy arm hair or whatever, you’re doing sports or something, that can really influence the outcomes [off the app]." (Depression) - "The problem is that another app somehow is also feeding me data, even in the GoogleFit, so I have twice the amount of steps every day. And that has blown things up, of course. I was well into the deficit range of calories that I could take in. That’s not quite accurate yet […]." (Obesity) |
| **Lack of (added) value** | - "Failure to improve health […] "doesn't help me, I have to try something else"" (Tinnitus) |
| **Lack of therapeutic setting** | - "That's a hazard when you implement something like that without a professional background. I see that as-, can be a problem."" (Cancer) - Yes, for example, it was relatively clear to me that the app alone would probably not be enough. (Depression) - "So I think it's also a little dangerous, in quotes, to just rely on the app now." (Obesity) - "For me, the problem is more like... There has to be someone behind it to watch what I'm doing." (Obesity) |
| **Patient Safety** | - "Well, I even had a psychotherapist once who advised me against it, because this continuous confrontation with the pain was supposed to push it into the subconscious […]" (Migraine) - "Such an app is not helpful. It probably throws the person quite far back, because he then suddenly gets things […] as if he was already half dead." (Alcohol dependence & Cancer) - "Yes, or when I realize this is not doing me any good. I have other aches and pains now, probably because I did the exercise wrong. Then I would also say: "You didn't have that before. Now you've done the exercise and it's gotten worse." Then I would also stop. (Osteoarthritis) - "[...] if there would have been triggers for me again. If then triggers would have arisen again, which would have given me […] a step backwards." (Different F-diagnoses) - "Many people don't want to talk about it [the disease] and withdraw. And I think that if you only have this app in front of you and nothing personal at all, that's very difficult for cancer patients. For all seriously ill people." (Cancer) - "We then type in a few letters and hope that we might find an answer there, even psychosocially, but talking to a person personally, that's completely different." (Cancer) - ""Suicidal thoughts" entered, simply as a test, not because it has been like that, and that is indeed a weak point, I have also discussed that with the marketer and said "Man, that is […] in terms of comorbidities actually, does that happen sometimes, depression and when it gets really bad with the tinnitus, there is nothing happening"" (Tinnitus) - "Or at some point I had indicated that I was having suicidal thoughts. And only then did the app unlock that you can call the telephone counselling service directly from the app." (Depression) - "So there would have to be a correction option somehow, otherwise you do too much compensatory movement. And you think you're the world champion and yet you didn't do the exercise right." (Osteoarthritis) - "[...] if someone has no idea at all or, as you also just said, then such mistakes creep in quickly and no one sees that, no one can control that and then you can do more wrong than anything else." (Osteoarthritis) |
| **Usability** | |
| **Problems with the instructions** | - "I would say, from my experience, I really don't have the nerve to read it at that moment. [...] But you could do it quite simply, like a how-to with screenshots. So rather with pictures you have to click on." (Migraine) - "So, that I don't have to search myself, but, that maybe you can also watch a little video or something that explains to you where are which buttons and what do I have to press to make it work." (Depression) |
| **Difficulties with the usage** | - "But then there's kind of no main menu after that. So when I finish it, I would like to have a main menu." (Migraine) - "And from there I thought, "That was definitely not well presented in the app if it was not understandable at all. And I do think that's a point for older people […] groups of 55 plus people with." (Migraine) - "And that it's also easy to find. There are also some apps where you search a lot before you have found what you actually wanted." (Obesity) - "So, that I don't have to search myself, but, that maybe you can also watch a little video or something that explains to you where are which buttons and what do I have to press to make it work." (Depression) |
| **Technology** | |
| **Problems with the software** | - "Unless […] [the app] freezes or you kind of get kicked out because something expired [then it's a problem] […]" (Tinnitus) - "And it was programmed in a very very poor and annoying way" (Tinnitus) - "If it catches a virus [it would be a problem]" (Cancer) - "Exactly, for me it was via browser. And it would actually have been easier if it had been done via an app, because at that time I did a lot of things on my cell phone, and it was always a bit confusing on the go. You always had to zoom in, zoom out." (different F-diagnoses) |
| **Problem with the hardware** | - "I think that using it would be even easier if I could sort of just talk to the phone and not have to sit down and look at the display the whole time." (Migraine) - "For me, this sensitivity to light was always quite extreme [...]. And then such a bright lit screen during a migraine attack is like poison for me." (Migraine) - "So I wish I could install them on two devices, because with the cell phone it's sometimes quite small with the reading that I would have it […], on the tablet as well." (Obesity) - "If you think about possible accessories, for example. I'm kind of getting out of the game with any more technical accessories. So I don't use a smartwatch, nor do I have VR goggles, and I don't really want to." (Cancer) - "Personally, I like it when you don't need anything additional for it. So I wouldn't want to buy a smartwatch. I wouldn't want to buy VR glasses either." (Depression) - "And technically is-, so maybe my phone got an update, the app doesn't work anymore, maybe only bad." (Depression) |
| **Problems with interoperability and network connection** | - "Next thing is what was brought up earlier, connection with any sports equipment. I just looked, there are ten different things in there. GoogleFit is also included, which I actually also use. Problem is, somehow, another app is also adding data, also into the GoogleFit, so I have twice the amount of steps every day." (Obesity) - "And that I-, we also had a bad Internet connection there, so I couldn't use it at all like that in some cases. (Tinnitus)" |
| **Unspecific technical problems** | - "[...] that when you're not well, when you're not fit, these technical complexities are really a problem." (migraine, osteoarthritis) |
| **Use and adherence** | |
| **Problems due to the attitude of users** | - "Yes, of course it always depends a bit on the people who use it. [...] If someone doesn't want to do it in advance, or is not fully committed, he shouldn't have something like that prescribed." (Tinnitus) - "The second thing is that I don't want to do therapy on digital devices because as a journalist I'm on the tablet, cell phone and laptop all day." (Tinnitus) - "Yeah, I think that's going to be a problem because they're basically going to have to get involved with the technology." (Alcohol & Cancer) - "Yes, maybe you don't have the motivation to do it alone. Many need the group to do the exercises." (Osteoarthritis) - "But I will say, there are people there who […] need much, much, much more drive. And they depend on the doctor to address that." (Depression) |
| **Problems that occurred in the context of usage** | - "[…], social context, when you're working full time and you're doing household and stuff, and in the evening-, maybe other things were also more important to me than still finishing the mindfulness-based stress management course." (Depression) - "Yes, it's challenging to take the time. So I actually had to learn that as well […]. But then that's basically an overcoming for me, to withdraw and have time for myself and to respond to my needs." (Depression) - "Yes, I have another idea. And that is if it accompanies us through the day, but does not determine our day, with its effort." (Obesity) - "[A reason to quit would be if] the app dictates what to do, "Now at eight o'clock, this has to be done."" (Cancer) - "And the last point I had was whether you can also use this app secretly. So to speak, if I'm sitting on the train and don't have any speakers with me. Whether they're always so low-threshold, so to speak, that you can use it to bridge time in the waiting room or something." (Depression) - "And I can't do that in front of the running TV, when family is sitting around, of course that doesn't work. And if someone says: "I don't have the time, because I have small children or we have a lot going on at home or something"-. So you do need a bit of a place to retreat." (Tinnitus)" |
| **Inadequate, unappealing content design** | - "For me, […] I didn't find it very-, so it had become somehow boring for me. So I didn't enjoy entering my information there anymore, because it was always the same-, It always just recorded the app and then calculated it for me and that was it." (Obesity) - "So I think there could be a few more relaxation exercises, that you have a little bit of variety, but it's definitely a good base already." (Migraine) - "[...] heard everything before, and I've heard it 30 times and there's no new content coming at all, it doesn't keep me happy or it doesn't motivate me to dial in anymore, then I also think I would switch probably." (Depression) - "Many people don't want to talk about it and withdraw. And I think that if you only have this app in front of you and nothing personal at all, that's very difficult for cancer patients. For all seriously ill people." (Cancer) - "[...] my husband would sit down and he would say, "I'm talking to a device. So what's that supposed to tell me?" (P5: The emotional component is missing, isn't it?) Right. So the emotional component is missing. There would-, there are many who say they don't like to talk about this disease either and certainly not in this way." (Cancer) |
| **Limited time resources** | - "[...] that's why I didn't use it any further, because it was also too extensive for me." (Migraine) - "First and foremost, no calorie counting, so I’m not entering data all day like on Weight Watchers." (Obesity) - "It's just very time-consuming, and that's the thing that bothered me a little bit before." (Migraine) - "And the amount of time required is, of course, relatively large. So I don't always manage to do everything I would like to. And you really have to consciously make the time and really be able to withdraw." (Tinnitus) - "So that's just important, that you can pause this video, which is shown there. The doorbell could be ringing, or something else, so it doesn't continue and you can pause it accordingly." (Arthrosis) |
| **Data privacy and security** | |
| **Lack of data security and data protection** | - "If I have the feeling the data is not in good hands [...] I do turn my innermost thoughts inside out with these apps. Especially when it comes to mental illness." (Depression) - "[…] I wouldn't have used the app, for example, if I was afraid-, if I had to fear that my data was somehow not secure and disappearing down some dark channels." (Depression) - "[...] if I now have to prove my sporting activities to the health insurance company, so be it, but they just get the sporting activities, but they are not supposed to know, how I felt. [...]" (Obesity) - "Privacy [may argue against the use]" (Cancer) - "The biggest problem I would have with such a thing is actually the data security. Because if I have an Android system with a corresponding smartphone, then[…] the data octopus called Google taps into things that run in the background. We all don't even know what actually ends up on Google's servers from our activities. That's the biggest problem for me, actually." (Cancer) |
| **Patient-Physician relationship** | |
| **Problems with usage not accompanied by a doctor or therapist** | - "[…] an app has limits, I think that's important and apps should be… and that should also, when the doctors eventually know what that is, also tell the patients (laughs), "Yes, that's just an app and it has its limits.""" (Depression) - "On the other hand, I've thought or fantasized around that if it's too individual, then maybe the person will slack because they'll think, "Oh, I don't need the personal contact at all. Or cancel on the psychologist or something." (Depression) - "Yes, for example, it was relatively clear to me that the app alone would probably not be enough." (Depression) - "That's a danger when you implement something like that without a professional background. I see that as-, can be a problem."" (Cancer) - "So I think it's also a little dangerous, in quotes, to just rely on the app now." (Obesity) - "With me, the problem is more like. There has to be someone behind it to check what I'm doing." (Obesity) |
| **Negative influences on the doctor-patient relationship** | - "It would bother me if I had to share the account with my doctor. (P4: Not me)" (Osteoarthritis) - "Because, of course, it must be assumed that the prescribing physician or the physician I’m attending is also behind it. If he now inwardly rejects it, then of course the relationship with him can suffer." (Tinnitus) - Doctors have actually already expressed scepticism, I have noticed, regarding this app. I don't know, if I have a problem and the doctor says 'I can't help you with this' and pushes everything off the edge that I otherwise bring with me in terms of possible solutions, then I should change doctors and not listen to what the doctor says there." (Tinnitus) - "if I would find out that he then says "Well, you're using your app, then you don't need me at all anymore, then do your thing there", I don't know, that would be a wrong reaction from a doctor." (Tinnitus) |
| **Divergent opinion between doctor and app** | - "Do I primarily trust my doctor and what he says? Or do I also additionally trust what an app suggests I might do? That's where I stand in between." (Cancer) |
| **Knowledge and skills** | |
| **Expandable skills, knowledge and experience of patients** | - "I can't operate at it all, I can't operate at it all. I'm not a person who can handle digital things at all." (Cancer) - "Otherwise, it may just be that the person just can't cope technically." (Depression) - "[...] how do you want to capture media literacy when someone first has installed an app. That's difficult to realize and certain things then require media literacy and if that's just starting up the app […]." (Tinnitus) |
| **Perception** | - "[...] I've already passed the 60, we just didn't grow up with these things in our hands. So maybe we just don't want to be digital for a change, and the use of paper is just more familiar." (Migraine) - "It kind of wasn't my product. Probably because it's digital, so anything I use for mental health is really just PMR or autogenic training. I've-, I think I've always been the 'analog guy' there." (Depression) - "I also think it's also questionable whether all older people would even be the target audience, at least those who really like to use pen and paper. I think they can continue to do that. I don't think you have to be forced to do something like that, and that's why I have complete understanding." (Migraine) - "Of course, you can-, you're not as nimble with it as younger people are now when you reach a certain age. Because you do-, yes, you do use it, but sometimes you do get stuck." (Tinnitus) - "That's where I'm somewhere in a tension and that's where I then lack confidence in the efficacies of a digital app like this." (Cancer) - "Psychology and app, that's such a bit-, I can't really imagine that yet." (Cancer) - "[...] if my husband sat down with it, he would say, "I'm talking to a device. So what's that supposed to tell me?"" (Cancer) - "Yes, I also think that not everyone is ready, to already work with this app. That they say, "This is not someone I'm sitting across from." So, (P6: Is nothing personal) right. It's too impersonal. (Cancer). - "I haven't used an app until now, because I always imagined it to be very complicated, I have to read a lot, scroll." (Obesity) |
| **Expandable skills, knowledge and experience of health care providers** | - "I think it would make a lot of sense. So you would just have to somehow bring it to the doctors a bit, because I think most of them don't really know how to deal with it yet." (Depression) - "Unfortunately, I think doctors often don't have the knowledge because they just don't have the time to dig into it" (migraine). - "For me, at least, I was the first one to get an app prescribed. I had to explain to the doctor and the health insurance company what it was (laughs) and whether or not they could prescribe it for me." (Depression) |
| **Individuality** | |
| **Inadequate adaption to individual user abilities and needs** | - "It’s so individual, of course. Everyone has their own problems of course; everyone perhaps does their own exercises" (Osteoarthritis). - It has to be user-friendly, and also, I'll say for slightly older cohorts, because you're not as skilled at using it as the younger generation is now." (Tinnitus) - "Especially also geared to when you just also have a bit more weight, that you can't do, what do I know, a 5km run right away, that that would just be adapted." (Obesity) |
| **Too generalized approach** | - "Tinnitus symptoms are very individual, both in terms of the triggers and the expression. And dealing with it and then the question is always, if such an app comes as a therapy concept, how fine-tuned is that [...]" (Tinnitus) - "In the end, it always has to remain an app where you're aware that it's written and invented for many people. And that individuality simply has a limit, and that's a good thing. Because otherwise I would drop other things. In the self-help group or whatever, where I go." (Depression) - "I would certainly have dropped out if I had the impression it was all done according to a pattern and that the personal entries were not taken into account. So if, what I state about myself, if that would not have been taken into account." (Depression) |
| **Implementation** | |
| **Barriers to access** | - "[…] not making complicated requests to the health insurance company, preferably something else in writing and you fax something […]." (Depression) - "That would be a desirable thing. I have also not asked whether that is possible to install yet. But I suspect not, because the activation code works once for a device and that’s it, which you get from the health insurance." (Obesity) - "My family doctor had also written them down as prescription and then I got a note from the health insurance company saying that my BMI was too high, so I couldn't get […] [the app]. It would have to be under 40. So, as I said, that was out of the question for me." (Obesity) - "For some, it is. For me it is not necessary, but-. There are enough migrants who are also seriously ill and who could not use something like this, even if it was recommended by the doctor that they should do it. (M1: Because the language is not provided all?) Because the language is not there." (Cancer) - "For me, as a pretty much blind person, it would definitely have to be barrier free." (Obesity) - "So, yes, it has to be low-threshold and if it's not, I think it's also difficult for a certain age group to deal with it or also for a certain degree of illness." (Depression) - "I also find that now in the sense of […] [the app], that there is really only a small target group addressed. So just really in this age group and I also find rather feminine." (Depression) |
| **Additional burden** | - "And whether they [the doctors] would have the time to do it, because nowadays it's difficult to get an appointment with a doctor." (cancer) - "Because that's another new task for the doctors. First of all, they have so much to do with their patients, who are all individuals, focused on their needs. So it becomes difficult to bring it all together afterwards. Because there won't be just one patient who wants to use such an app, but five or even 100." (Cancer) |
| **Low acceptance by health care providers** | - "Myself, I'm not-, not an active user of any of these headache apps because, for once, I'm thinking about my neurologist, who's still so close to retirement and I don't think he wouldn't be that interested if I would show up with an app there now." (Migraine) - "And our doctor, [...] who is not old yet, totally rejects something like that. She sees it somehow as a personal interference in her competence, [...]. And that's very difficult that some doctors feel attacked there." (Cancer) - "That might have depended on the emphasis of the rejection as well. Just a "Yeah, well, you can do it, I don't know." is maybe something different than, "By all means, don't do that, it's nonsense!"" (Depression) - "Do I go to my urologist, do I go to my thoracic surgeon, do I go to my family doctor, or do I go to my dermatologist? And if one is in favour, but the others are against, what do I do?" (Cancer) - "Yeah, I guess if the doctor says, "Don't do it, it's not good for you," then I just don't do it. " (Osteoarthritis) - "It's not as accepted by some physicians. This digitization is a great thing, but I think some doctors, they reject something like that and don't want that." (Cancer) - "Maybe it's also a conflict of interest, because the providers of the app probably want to make money with it. They earn money with it and therapists also earn money somehow. And maybe they just don't necessarily want to make it into advertising for one or the other" (Depression). |
| **Difficult transfer into clinical practice** | - "Because just no one has recommended this app yet. So I've really been through a bunch of psychologists and I-, several clinics [...]. But the topic of the app is not in the waiting room with a flyer, nor at the doctor's office, nor in the self-help group [...]" (Depression) - "It could really be more public. That right there would be another thing. Because few people know that this list exists and what you can get there." (Tinnitus) |
| **Too many options to choose** | - "I'm struggling with my illness or I'm struggling with my various illnesses, and then now there's this additional psychological pressure: "I might have the wrong thing I'm using after all. Maybe there's something significantly better in the meantime." These digital health apps, they're totally confusing to me as a user." (Cancer) |
| **Fear of consequences due to app usage** | - "What does that do to my health insurance coverage afterward?" (Cancer) |
| **Costs** | |
| **Loss of revenue for health care practitioners** | - "[For the doctor it is] of course [...] a loss of revenue if I use such a thing." (Osteoarthritis) |
| **Low willingness to pay** | - "So for me, the reason was that it's paid now and that-, so this app was super good, except for some initial difficulties, but just, I didn't want to pay for it" (obesity) - "So I don't want to be prompted by an app like this into having to buy any additional accessories." (Cancer) |
| **Alternative financing methods** | - "If, for example, advertising were to come on all of a sudden. If [advertisements] from different drug manufacturers would always be shown there." (Cancer) - "It could just as well be the digital world seeing that the woman or the man is interested in health apps. Then I get some kind of influx like that all the time. I don't want that." (Cancer) |
| **Waste of money** | - "But the control was there, otherwise I think that's partly throwing money out the window when it’s practically paid for and people say, "Oh I'll take a look and I'll go and see.""" (Osteoarthritis) - "And it was, that was also point of attack, of those who do not think anything at all of e-health, that the scientific proof is missing and now the insurance already pays" (Tinnitus) |
